# Inhibitory activity of flavonoids fraction from *Astragalus membranaceus* Fisch. ex Bunge stems and leaves on *Bacillus cereus* and its separation and purification

**DOI:** 10.3389/fphar.2023.1183393

**Published:** 2023-07-03

**Authors:** Liyan Cui, Zhennan Ma, Wenhui Li, Haihui Ma, Shang Guo, Defu Wang, Yanbing Niu

**Affiliations:** ^1^ College of Grassland Science, Shanxi Agricultural University, Jinzhong, Shanxi, China; ^2^ College of Life Sciences, Shanxi Agricultural University, Jinzhong, Shanxi, China; ^3^ Shanxi Institute for Functional Food, Shanxi Agricultural University, Taiyuan, Shanxi, China

**Keywords:** *B. cereus*, *A. membranaceus* stems and leaves, flavonoids, antibacterial activity, molecular docking

## Abstract

**Introduction:** *Astragalus* *membranaceus* Fisch. ex Bunge is a traditional botanical drug with antibacterial, antioxidant, antiviral, and other biological activities. In the process of industrialization of *A. membranaceus*, most of the aboveground stems and leaves are discarded without resource utilization except for a small amount of low-value applications such as composting. This study explored the antibacterial activity of *A. membranaceus* stem and leaf extracts to evaluate its potential as a feed antibiotic substitute.

**Materials and methods:** The antibacterial activity of the flavonoid, saponin, and polysaccharide fractions in *A. membranaceus* stems and leaves was evaluated by the disk diffusion method. The inhibitory activity of the flavonoid fraction from *A. membranaceus* stems and leaves on *B. cereus* was explored from the aspects of the growth curve, cell wall, cell membrane, biofilm, bacterial protein, and virulence factors. On this basis, the flavonoid fraction in *A. membranaceus* stems and leaves were isolated and purified by column chromatography to determine the main antibacterial components.

**Results:** The flavonoid fraction in *A. membranaceus* stems and leaves had significant inhibitory activity against *B. cereus*, and the minimum inhibitory concentration (MIC) and minimum bactericidal concentration (MBC) were 1.5625 and 6.25 mg/mL, respectively. *A. membranaceus* stem and leaf flavonoid fraction can induce death of *B. cereus* in many ways, such as inhibiting growth, destroying cell wall and cell membrane integrity, inhibiting biofilm formation, inhibiting bacterial protein synthesis, and downregulating virulence factor expression. In addition, it was clear that the main flavonoid with antibacterial activity in *A. membranaceus* stems and leaves was isoliquiritigenin. Molecular docking showed that isoliquiritigenin could form a hydrogen bonding force with FtsZ.

**Conclusion:** *A. membranaceus* stem and leaf flavonoid fractions had significant inhibitory activity against *B. cereus*, and the main chemical composition was isoliquiritigenin.

## 1 Introduction

*Bacillus cereus* is a type of gram-positive bacterium that produces defensive endospores and causes beta-hemolysis without any specific geographic distribution. *Bacillus* species are omnipresent in nature due to the simplicity of the nutrients they require to grow (Tewari et al., 2015). The natural hosts of *B. cereus* embrace decaying organic matter, water, vegetables, pollutants, feed, and intestinal flora of different animals. It is one of the three major pathogens causing mastitis in dairy cows (Kumari and Sarkar, 2016). *B. cereus* also colonizes and adheres to breast epithelial cells faster with other pathogens as vectors, thereby accelerating the disease and even causing death in cows (Döpfer et al., 2000). Cows infected with *B. cereus* exhibit decreased milk production and loss of lactation ability, inflicting serious losses to the economic development of animal husbandry worldwide (Wu et al., 2009). Soil, feed, and bedding are the main sources of *B. cereus* infection in dairy cows, and feed is the only source of *B. cereus* spores during the captive period of dairy cows (Kumari and Sarkar, 2016).

With prohibition and restriction on the employment of antibiotic additives in many countries, the research and development of alternative technologies and products that can effectively replace the growth promotion and disease prevention effects of antibiotics in livestock and poultry breeding have become a research hotspot in the current animal husbandry industry. Plant-derived feed additives have attracted increasing attention (Guo et al., 2020; Jambwa et al., 2021). Among them, traditional botanical drug feed products based on natural resources have become a research hotspot of antibiotic substitutes for feed products due to their immune enhancement, anti-microbial, digestion, and growth promotion functions (Yin, 2018; Ding et al., 2019).

*Astragalus membranaceus* Fisch. ex Bunge (Fabaceae; *Astragalus membranaceus* radix et rhizoma) is one of the more than 18,000 species in about 650 genera of *Leguminosae*. This genus is mainly used for producing animal feed, followed by medicinal and green fertilizers. In China, its root is a commonly used bulk medicinal material that nourishes the Qi. As a result of its rich polysaccharide, flavonoids, and saponins content, it exhibits immune regulation (Wang et al., 2009), hypoglycemic (Chan et al., 2009), anti-inflammatory (Choi et al., 2007), antioxidant (Li et al., 2010), and antiviral activities. In 2012, the Ministry of Agriculture and Rural Affairs of the People’s Republic of China included it in the Catalogue of Feed Raw Materials (Announcement, 2012). In 2018, the Chinese Health Commission included it in the Catalogue of Medicinal and Edible Chinese Herbal Materials (Announcement, 2018). *A. membranaceus* stems and leaves are often discarded as a by-product of the *A. membranaceus* industry. Research has shown that *A. membranaceus* stems and leaves are also rich in active substances (Zhang et al., 2016; Li et al., 2020; Cui et al., 2022).

As a kind of phenolic metabolite, flavonoids are a class of secondary metabolites produced by plants. The content of flavonoids depends on the degree of lignification of plants. They are usually a series of compounds (C_6_-C_3_-C_6_) formed by two benzene rings containing phenolic hydroxyl groups through the central three-carbon chain (He, 2014). Flavonoids can be classified into flavones, flavonols, chalcones, isoflavones, dihydroflavonoids, dihydroflavonols, and others according to the characteristics of the linkage location of the B ring, oxidation extent of the central three-carbon chain, and formation of the ring (Li, 2012). The antibacterial activity of flavonoids is related to their structure. Studies have shown that the hydroxylation of flavonoids can affect their antibacterial activity (Yang et al., 2013). The double hydroxylation structure of dihydroflavone B- or A-ring is very important for the inhibition of methicillin-resistant *Staphylococcus aureus* (Tsuchiya et al., 1996). 2,4,2′-trihydroxy-5′-methylchalcone and 2,4,2′-trihydroxychalcone have inhibitory activity against a variety of *Streptococcus mutans* (Sato et al., 2010). The long-chain fatty substituents on the A-ring also affect the antibacterial activity of flavonoids. Stapleton et al. (2004) showed that the substitution of the sixth or eighth position of flavan-3-ols with C8 and C10 chains can improve the inhibitory activity against *Staphylococcus*. In addition, the halogenation of flavonoids also affects their antibacterial activity. Studies have shown that B-ring halogenation enhances antibacterial activity, while A-ring halogenation reduces antibacterial activity (Alcaraz et al., 2000). Flavonoids have important therapeutic and preventive functions such as protecting cardiovascular health, anti-oxidation, anti-tumor, and regulating immunity. It has become a hotspot in the development and utilization of natural medicines, feed substitutes, and healthcare products at home and abroad (Zhang et al., 2008; Li et al., 2015).

The purpose of this research was to assess the antibacterial activity of *A. membranaceus* stem and leaf extracts and explore their inhibitory mechanism against *B. cereus* to evaluate its prospect for the development of feed additives.

## 2 Materials and methods

### 2.1 Materials and reagents

The stems and leaves of *A. membranaceus* were collected from Fangshan City, China, in July–August 2021. The plant's name was confirmed in a website (http://powo.science.kew.org/taxon/urn:lsid:ipni.org:names:478611-1). All the plant material collected was washed clean with tap water, air-dried at room temperature, and ground into a powder with a pulverizer. Beef extract peptone agar, beef extract peptone, chloramphenicol, Coomassie Brilliant Blue R-250, and Sephadex LH-20 were purchased from Solarbio (Beijing, China). A micro alkali proteinase assay kit was purchased from Elabscience (Wuhan, China). Crystal violet was purchased from Sangon Biotech (Shanghai, China). The TransZol Up Plus RNA Kit was purchased from Trans (Beijing, China). The PrimeScript™ RT Kit was purchased from Takara Bio (Dalian, China). All other chemicals and solvents used were of analytical grade. Isoliquiritigenin (Cat No. SI8220), biochanin A (Cat No. SB8240), and isorhamnetin (Cat No. SI8280) were purchased from Solarbio (Beijing, China), and the purity of the three standards was ≥98% by high-performance liquid chromatography (HPLC).

### 2.2 Test strains and sources

The tested strains were all from the China Center of Industrial Culture Collection, which included gram-positive bacteria (*B. cereus* and *Staphylococcus aureus*) and gram-negative bacteria (*Escherichia coli*, *Salmonella enterica* subsp. enterica, *Shigella flexneri*, *Cronobacter sakazakii*, and *Shigella dysenteriae*). All bacterial strains were stored at −80°C and activated by overnight incubation in beef extract peptone broth at 37°C and 170 rpm.

### 2.3 Extraction of flavonoid, saponin, and polysaccharide fractions from *A. membranaceus* stems and leaves

The flavonoid, saponin, and polysaccharide fractions from *A. membranaceus* stems and leaves were extracted using the method of Cui et al. (2022). In brief, powdered plant material (1.5 kg) was subjected to ultrasound-assisted extraction using 75% ethanol to obtain a crude extract. The crude extract was partitioned by dissolving in water. It was then partitioned into petroleum ether, ethyl acetate, and n-butanol to obtain the petroleum ether fraction (PEF), ethyl acetate fraction (EAF), and n-butanol fraction (BF), respectively, and the aqueous fraction (AF). EAF and BF were eluted with different ethanol concentrations (0%, 30%, 50%, 70%, and 95%) using AB-8 and D101 macroporous resins. The 50% ethanol elution comprised the flavonoid and saponin fractions. The polysaccharide fraction was obtained by water extraction and alcohol precipitation.

### 2.4 Antibacterial activity of *A. membranaceus* stem and leaf extracts

The antibacterial activity of *A. membranaceus* stem and leaf extracts was determined by the disk diffusion method (Fei et al., 2019). Bacteria-containing solid plates were prepared by pouring 20 mL of the beef extract peptone agar and 100 μL of bacterial suspension (OD_600_ = 0.5). A filter paper of 6 mm diameter was placed on the prepared bacteria-containing plate, and 15 μL of the flavonoid, saponin, and polysaccharide fractions (10 mg/mL) was placed on the filter paper. The plate was then incubated at 37°C for 24 h. The same solvent was used as the control. The inhibitory activity against different strains was assessed by the diameter of the inhibition zone.

### 2.5 Minimum inhibitory concentration and minimum bactericidal concentration of flavonoid fraction from *A. membranaceus* stems and leaves against *B. cereus*

The twofold dilution method was used to determine the minimum inhibitory concentration (MIC) of the flavonoid fraction against *B. cereus* (Liang et al., 2020). In brief, bacterial suspensions (OD_600_ = 0.5) were prepared by inoculating strains in the logarithmic phase into the liquid media. The flavonoid fraction was adjusted to final concentrations of 0, 0.390625, 0.78125, 1.5625, 3.125, 6.25, 12.5, and 25 mg/mL, with chloramphenicol (5 mg/mL) as the positive control. The bacterial suspension (1/100 V) was inoculated into the nutrient broth with different flavonoid fraction/chloramphenicol concentrations and inoculated for 24 h in a 37°C incubator. The MIC was the concentration without bacterial growth. About 100 μL of the culture was treated with ≥1 MIC of the flavonoid fraction to spread on the solid plates and incubated at 37°C for 24 h. The concentration with ≤5 colonies was defined as the minimum bactericidal concentration (MBC).

### 2.6 Growth curve of *B. cereus*

The growth curve of *B. cereus* was drawn according to the report of Shi et al. (2016). In brief, *B. cereus* was cultured until the absorbance at 600 nm of the bacterial solution reached 0.5 (OD_600_ = 0.5). The activated *B. cereus* was inoculated in 5 mL of the liquid medium and treated with different flavonoid concentrations (1/8 MIC, 1/4 MIC, 1/2 MIC, and 1 MIC). The normal cultured *B. cereus* was used as the control. Cultures were grown at 37°C and 170 rpm. Growth was measured as OD_600_ every 2 h using a multifunctional plate reader.

### 2.7 Effect on cell wall of *B. cereus*

Alkaline phosphatase (AKPase) is an enzyme that exists between the cell wall and cell membrane. Under normal circumstances, it will not be released to the outside of the cell. However, when the integrity of the cell wall is destroyed, AKPase will be released from the inside to the outside of the cell, such that the extracellular environmental AKPase activity is significantly increased. Therefore, the detection of extracellular AKPase activity can determine whether the integrity of the cell wall is damaged (Diao et al., 2018). AKPase content of *B. cereus* was measured with reference to a previously reported method (He et al., 2018). *B. cereus* was treated with different concentrations of the flavonoid fraction (1/2 MIC, 1 MIC, and 2 MIC). The samples were obtained every 2 h (or 1 h) and centrifuged at 8,000 rpm for 10 min. The AKPase content in the supernatant was measured with the Alkali Proteinase Assay Kit according to the manufacturer’s instructions.

### 2.8 Effect on cell membrane of *B. cereus*

The destruction of the bacterial cell membrane integrity causes the leakage of intracellular components, and the released amount of macromolecules such as nucleic acid and protein can be measured by the absorbance at 260 and 280 nm. These methods were performed according to the literature, and appropriate adjustments were made. The cell membrane integrity of *B. cereus* was determined with reference to previous reports (Wu et al., 2017; Zhang et al., 2020). Log-phase *B. cereus* was collected by centrifugation at 8,000 rpm for 10 min, and resuspended in 0.1 mol/L PBS (pH 7.2) to maintain its final density at OD_600_ = 0.5. The bacterial suspension was incubated with different concentrations of the flavonoid fraction (1/2 MIC, 1 MIC, and 2 MIC) at 37°C. The *B. cereus* culture without the flavonoid fraction was used as the control. After the mixture was filter-sterilized, the concentration of nucleic acids and proteins in the supernatant was measured with a microplate reader.

### 2.9 Effect of flavonoid fraction from *A. membranaceus* stems and leaves on *B. cereus* biofilm formation

The effect of the flavonoid fraction on *B. cereus* biofilm formation was analyzed using the crystal violet staining technique, as delineated by Andres et al. (2021). In brief, twofold serial dilutions containing nutrient broth and different concentrations of the flavonoid fraction (1/2 MIC, 1 MIC, and 2 MIC) were prepared. PBS was used as the control. After incubation at 37°C for 24 h, the cultures were washed thrice with PBS. After methanol fixation, 1% crystal violet staining solution was added to the well plates that were shaken gently to produce uniform staining. The plates were then washed with water, and 250 μL of 33% acetic acid was added to every well to dissolve residual crystal violet within the biofilm matrix. After 5 min of incubation, the absorbance (600 nm) was measured. The inhibition of biofilm formation was calculated using the following formula:

Inhibition rate (%) = (1 − OD_experimental_/OD_control_) × 100%.

### 2.10 Analysis of bacterial proteins by SDS-PAGE

Referring to Fei et al. (2019), the influence of the flavonoid fraction on *B. cereus* proteins was analyzed by using SDS-PAGE. Activated *B. cereus* (OD_600_ = 0.5) was treated with different concentrations of the flavonoid fraction (1/2 MIC, 1 MIC, and 2 MIC, respectively) at 37°C for 1 h. The cultures were collected by centrifugation at 5,000 rpm for 10 min and resuspended in sterile PBS (pH 7.4), and the bacterial proteins were obtained by ultrasound-assisted extraction for 30 min. The supernatants were obtained by centrifugation at 5,000 rpm for 10 min at 4°C. After mixing with SDS-PAGE loading buffer, the samples were placed in a 95°C water bath for 10 min and then analyzed by using SDS-PAGE, using 5% stacking gel and 12% separating gel. The protein bands were observed after staining with Coomassie Brilliant Blue R-250 staining solution. Untreated *B. cereus* was used as the negative control.

### 2.11 Influence on virulence genes via qRT-PCR

*B. cereus* strains were grown to the mid-log phase at 37°C in the nutrient broth with different concentrations of the flavonoid fraction (1/16 MIC, 1/8 MIC, 1/4 MIC, and 1/2 MIC). All samples were centrifuged at 5,000 rpm for 10 min to gather the pellet. Total RNA was extracted with the TransZol Up plus RNA Kit. First-strand cDNA was synthesized using 1 µg of total RNA according to the instructions of the PrimeScript™ RT Kit. The primer sequences are shown in Table 1. The reactions were performed at 94°C for 15 s, followed by 40 cycles of 5 s at 94°C and 30 s at 58°C. The results were calculated using the 2^−ΔΔCT^ method.

**TABLE 1:** Primers for qRT-PCR.

Primer names	Primer sequence (5′–3′)
*GroEL*-F	GAA​GAG​TCT​AAA​GGA​TTC​ACA​ACA​GA
*GroEL*-R	GGG​TTA​TCA​AGA​ACT​GCT​TCC​AT
*cytK*-F	CGG​TTT​CTC​TCC​TGG​TAT​G
*cytK*-R	AGC​CTG​GAC​GAA​GTT​GGT​A
*HblC*-F	AGA​AGG​GTT​TTC​GGA​TAG​AC
*HblC*-R	CGC​AGT​TGC​CAC​ATT​AGT​AT
*HblD*-F	ACA​GGC​AAC​GAT​TCC​ACA​AC
*HblD*-R	CCG​AGA​GTC​CAC​CAA​CAA​CA

### 2.12 Separation and purification of flavonoid fraction from *A. membranaceus* stems and leaves

**FIGURE 1:**
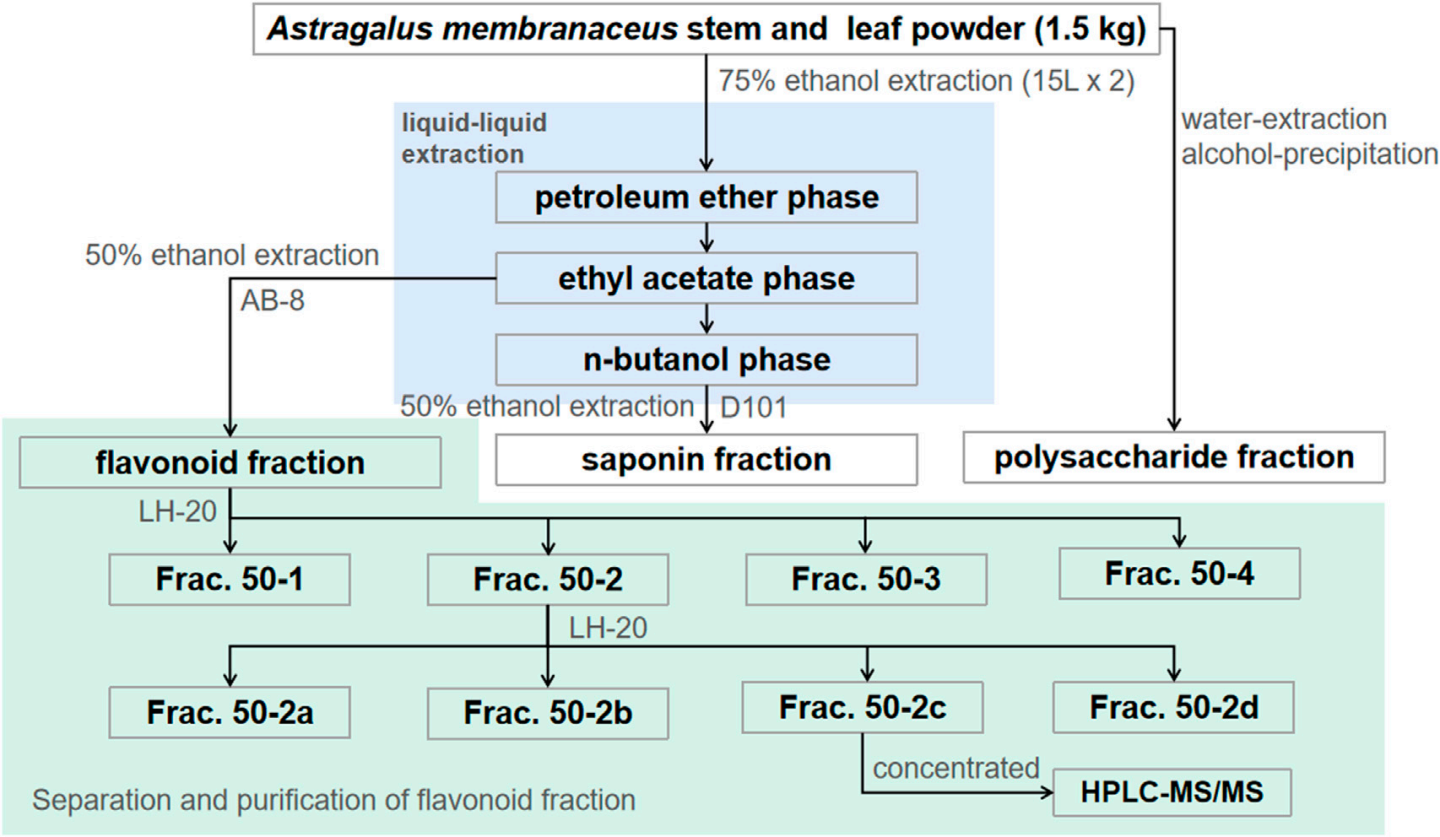
Separation and purification processes of *A. membranaceus* stem and leaf flavonoid fraction–guided antibacterial activity.

The flavonoid fraction from *A. membranaceus* stems and leaves was isolated and analyzed by AB-8 macroporous resin and Sephadex LH-20 column chromatography guided by antibacterial activity. The extraction method of the flavonoid fraction from *A. membranaceus* stems and leaves was the same as described in Section 2.3. The separation and purification processes are as follows: the EAF fraction (3.2 g) was separated by AB-8 macroporous resin and eluted with different concentrations of ethanol (30%, 50%, 70%, and 95%) to obtain four fractions (Frac. 30, 50, 70, and 95). Frac. 50 (1.269 g) was loaded on a Sephadex LH-20 column for chromatography to obtain four fractions (Frac. 50-1, 50-2, 50-3, and 50-4). Frac. 50-2 (0.266 g) was further subjected to Sephadex LH-20 column chromatography to obtain four fractions (Frac. 50-2a, 50-2b, 50-2c, and 50-2d). The fractions (about 15 mL each) were determined via thin layer chromatography (TLC) for merging. The inhibitory activity of each fraction against *B. cereus* was evaluated by using the disk diffusion method. The separation and purification processes of the flavonoid fraction from *A. membranaceus* stems and leaves are shown in Figure 1.

### 2.13 High-performance liquid chromatography–tandem mass spectrometry analysis

Following the procedures described by Cui et al. (2022) and Wang et al. (2021), the flavonoid composition of Frac. 50-2c was analyzed by the high-performance liquid chromatography–tandem mass spectrometry (HPLC-MS/MS) analysis. Identification of qualitative components was based on matching mass spectra with standard compounds available in the Thermo mzCloud online library and Thermo mzValut local library.

### 2.14 Verification test and molecular docking of isoliquiritigenin

The antibacterial activities of isoliquiritigenin, biochanin A, and isorhamnetin were evaluated by filter paper diffusion (5 mg/mL). On the basis of antibacterial activity, the UCSF Chimera software was used for the molecular docking of filamenting temperature-sensitive mutant Z (FtsZ: 4XSG) protein with isoliquiritigenin to predict its interaction force. First, FtsZ protein and isoliquiritigenin were pretreated, which included removing water molecules, adding hydrogen atoms, adding electrons, and minimizing energy. Second, the molecular docking of FtsZ and isoliquiritigenin was performed using the AutoDock Vina program to predict their affinity. The grid box with dimensions of 50 points × 55 points × 55 points was centered on the active site of the protein. Finally, the stability of FtsZ–isoliquiritigenin binding was evaluated with reference to the binding energy. A low score corresponded to high stability.

### 2.15 Statistical analysis

All measurements were performed in triplicate. The results are expressed as mean values and standard deviation. Experimental data were processed using GraphPad 8.0. Significant differences between the mean values were determined via the Duncan’s multiple range test. *p* < 0.05 was considered statistically significant.

## 3 Results

### 3.1 Antibacterial activity of *A. membranaceus* stem and leaf extracts

**FIGURE 2:**
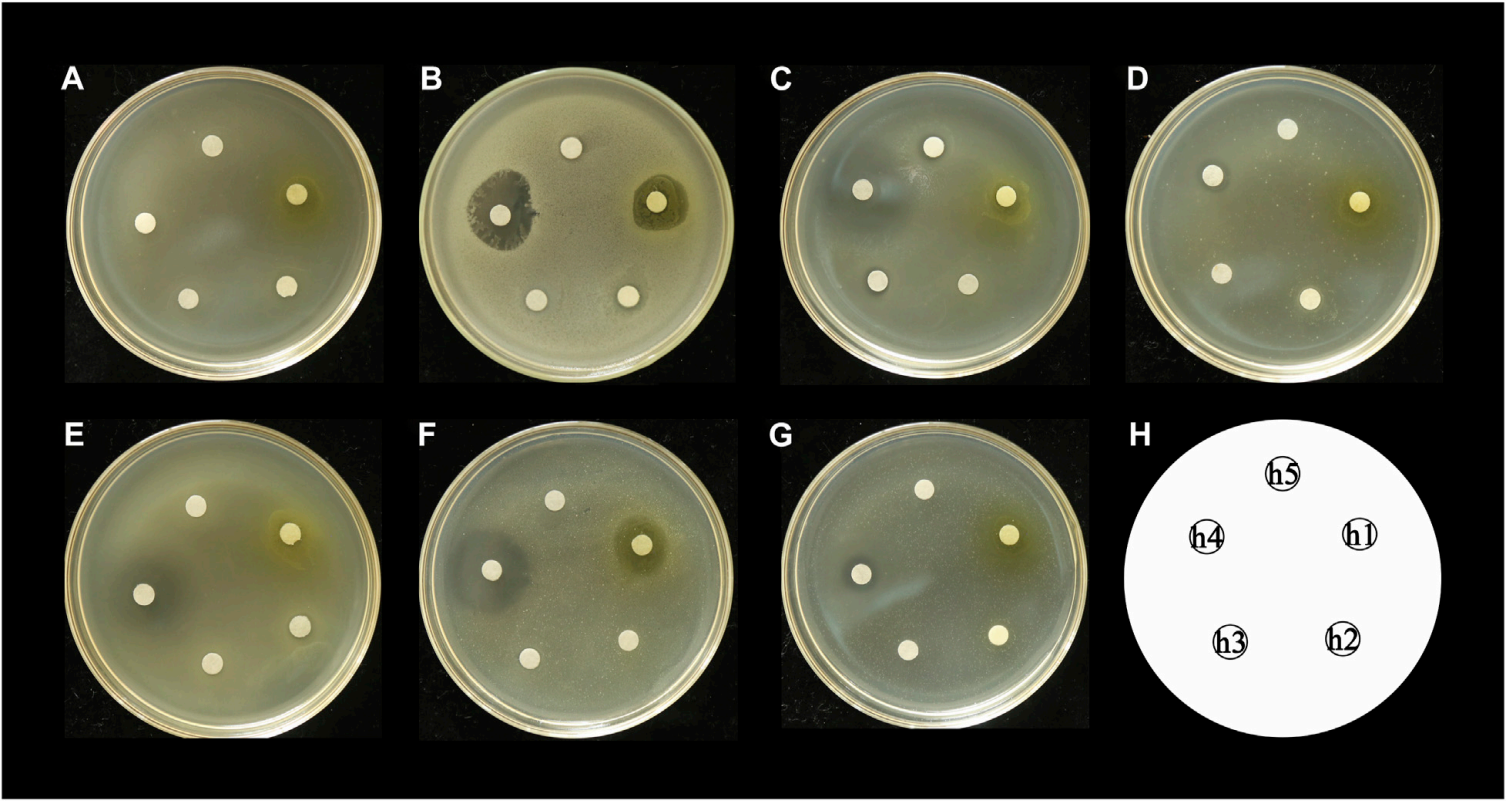
Inhibitory effect of *A. membranaceus* stem and leaf extracts on tested bacteria: **(A)** *E. coli*, **(B)** *B. cereus*, **(C)** *S. enterica*, **(D)** *S. flexneri*, **(E)** *C. sakazakii*, **(F)** *S. aureus*, and **(G)** *S. dysenteriae*; h1: flavonoid fraction; h2: saponin fraction; h3: polysaccharide fraction; h4: positive control; h5: negative control.

**FIGURE 3:**
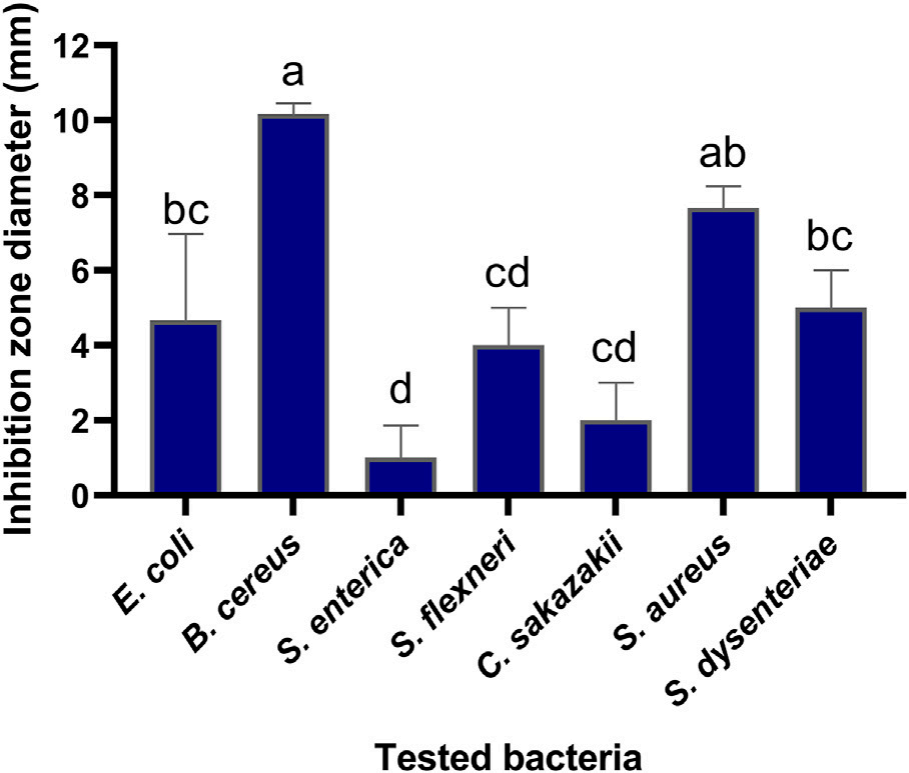
Inhibitory effect of flavonoid fraction from *A. membranaceus* stems and leaves on tested bacteria. Different letters indicate significant differences at the 0.05 level. (a: the inhibition rate of *B*. *cereus* was significantly different from that of *E. coli*, *S. dysenteriae*, *S. flexneri*, *C. sakazakii*, and *S. enterica*; ab: the inhibition rate of *S. aureus* was significantly different from that of *S. flexneri*, *C. sakazakii*, and *S. enterica*; bc: the inhibition rate of *S. dysenteriae* and *E. coli* was significantly different from that of *B. cereus* and *S. enterica*; cd: the inhibition rate of *S. flexneri* and *C. sakazakii* was significantly different from that of *B. cereus*, *S. aureus*, and *S. enterica*; d: the inhibition rate of *S. enterica* was significantly different from that of *B. cereus*, *S. aureus*, *S. dysenteriae*, and *E. coli*).

The antibacterial activities of the flavonoid, polysaccharide, and saponin fractions of *A. membranaceus* stems and leaves against selected bacteria were screened. Seven bacteria were evaluated for each extract in triplicate by the disk diffusion method at a concentration of 10 mg/mL. The results showed that *A. membranous* stem and leaf flavonoid fractions showed significant antibacterial activity when compared with the saponin and polysaccharide fractions, especially against *B. cereus* (Figure 2). The inhibitory effect of *A. membranaceus* stem and leaf flavonoid fraction on the tested bacteria is shown in Figure 3. The order of inhibitory activity against the seven tested strains is *B. cereus* > *S. aureus* > *S. dysenteriae* > *E. coli* > *S. flexneri* > *C. sakazakii* > *S. enterica*, and the difference was significant at *p* < 0.05 (Table 2). The diameters of the inhibition zones for bacteria in *B. cereus*, *S. aureus*, *S. dysenteriae*, *E. coli*, *S. flexneri*, *C. sakazakii*, and *S. enterica* were 10.167, 7.667, 5.0, 4.667, 4.0, 2.0, and 1.0 mm, respectively. Using the twofold dilution assay, the MIC and MBC of the flavonoid fraction against *B. cereus* were 1.5625 and 6.25 mg/mL, respectively. The flavonoid fraction from *A. membranaceus* stems and leaves showed the strongest inhibitory activity against *B. cereus*, and its possible antibacterial mechanism was further analyzed.

**TABLE 2:** ANOVA result for the antimicrobial activity of flavonoid fraction from *A. membranaceus* stems and leaves on the tested bacteria.

Source of variance	Square sum	Df	Mean square sum	F value	*p* value
Between treatment	181.8	6	30.30	19.58	<0.0001
In treatment	21.67	14	1.548		
Total variance	203.5	20			

### 3.2 Effect of flavonoid fraction from *A. membranaceus* stems and leaves on growth of *B. cereus*

**FIGURE 4:**
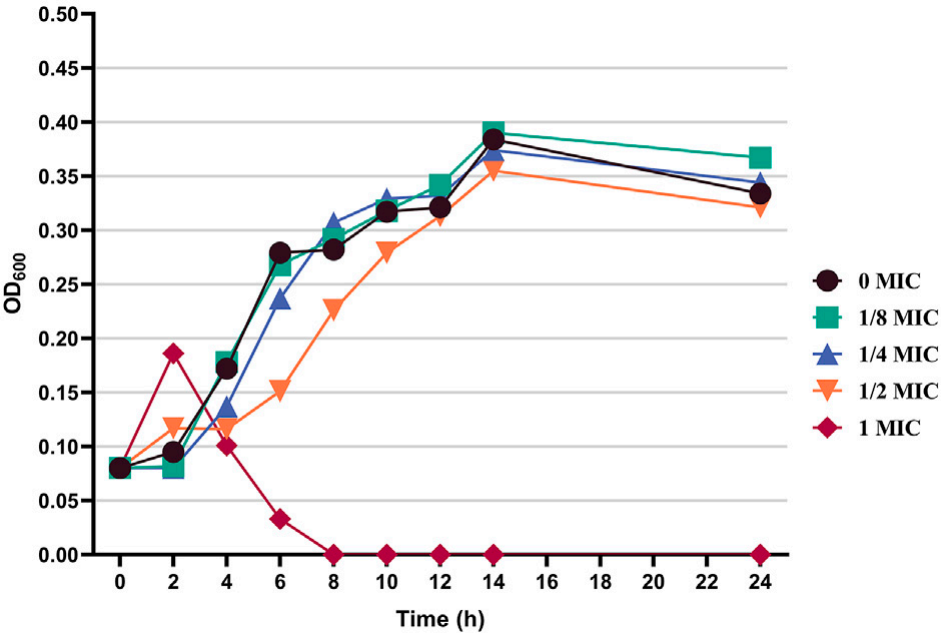
Effect of flavonoid fraction from *A. membranaceus* stems and leaves on the growth curve of *B. cereus*.

On the basis of the activity measurement results, *B. cereus* was selected as the test bacteria, and its antibacterial effect and inhibition were further discussed. Figure 4 shows the effects of different concentrations of the flavonoid fraction on the growth of *B. cereus*. When compared with the control group, the flavonoid fraction at the concentration of the MIC almost completely inhibited the growth of *B. cereus* within 8 h of incubation time. From the time–growth curve of *B. cereus*, the flavonoid fraction advanced its logarithmic phase. Within a certain concentration range, the bacteriostatic effect was enhanced with increase in the flavonoid fraction.

### 3.3 Effect of flavonoid fraction from *A. membranaceus* stems and leaves on cell wall and cell membrane of *B. cereus*

**FIGURE 5:**
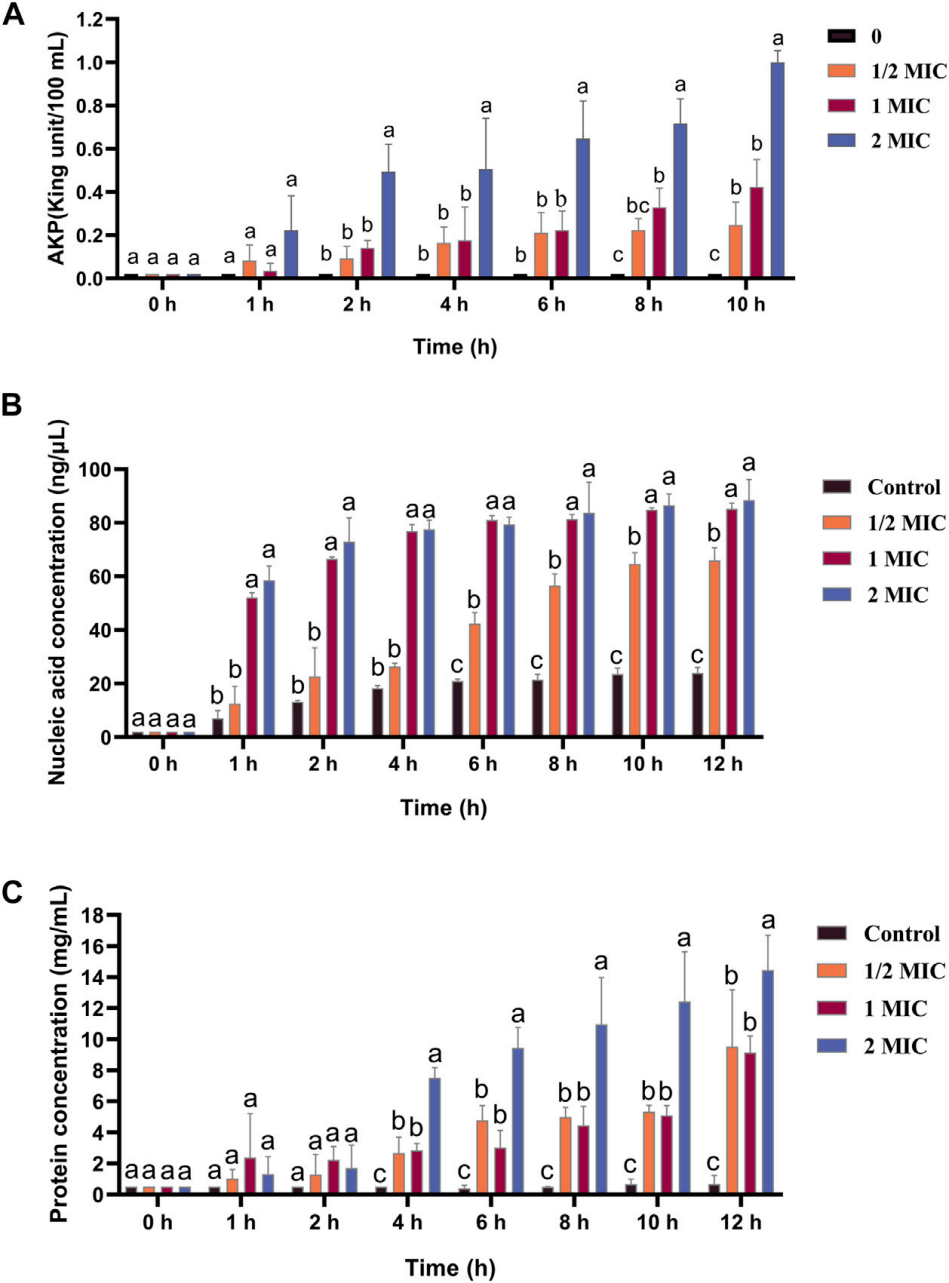
Effect of flavonoid fraction from *A. membranaceus* stems and leaves on the cell wall and cell membrane of *B. cereus*. **(A)** Extracellular AKPase concentration: 0 h and 1 h (a: no SDi between 1/2 MIC, 1 MIC, 2 MIC, and control); 2 h, 4 h, and 6 h (a: SDi between 2 MIC and control; b: no SDi between 1/2 MIC, 1 MIC, and control); 8 h (a: SDi between 2 MIC and control; b: SDi between 1 MIC and control; bc: no SDi between the 1/2 MIC and control; c: SDis between 2 MIC, 1 MIC, and control); 10 h (a: SDi between 2 MIC and control; b: SDis between 1/2 MIC, 1 MIC, and control; c: SDis between 2 MIC, 1 MIC, 1/2 MIC, and control). **(B)** Nucleic acid release: 0 h (a: no SDi between 1/2 MIC, 1 MIC, 2 MIC, and control); 1 h, 2 h, and 4 h (a: SDis between 2 MIC, 1 MIC, and control; b: no SDi between 1/2 MIC and control); 6 h, 8 h, 10 h, and 12 h (a: SDis between 2 MIC, 1 MIC, and control; b: SDi between 1/2 MIC and control; c: SDis between 2 MIC, 1 MIC, 1/2 MIC, and control). **(C)** Protein release: 0 h, 1 h, and 2 h (a: no SDi between 1/2 MIC, 1 MIC, 2 MIC, and control); 4 h, 6 h, 8 h, 10 h, and 12 h (a: SDi between 2 MIC and control; b: SDIs between 1 MIC, 1/2 MIC, and control; c: SDis between 2 MIC, 1 MIC, 1/2 MIC, and control). SDi = detected significant difference.

AKPase is an intracellular enzyme between the cell wall and cell membrane, and its activity can only be detected extracellularly when the bacterial cell wall is damaged. The extracellular AKPase activity after treatment with different concentrations of the flavonoid fraction is shown in Figure 5A. No significant change in AKPase activity was noted in the control group. After flavonoid treatment, AKPase activity increased with flavonoid concentration and culture time. When the concentration of the flavonoid fraction was 1/2 MIC and 1 MIC, the extracellular AKPase activity of *B. cereus* after 8 h of treatment was significantly different from that of the control group, and there was a significant difference between the 1/2 MIC and 1 MIC treatment groups. When the concentration of the flavonoid fraction was 2 MIC, the extracellular AKPase activity was significantly different from that of the control group after 2 h of treatment. The results show that the flavonoid fraction disrupted the integrity of the *B. cereus* cell wall.

The nucleic acid and protein released by treatment with different concentrations of the flavonoid fraction are shown in Figures 5B, C, and the control group did not show any significant changes. When the bacteria were treated with the flavonoid fraction, the OD values at 260 and 280 nm increased significantly (*p* < 0.05), and the content of extracellular nucleic acids and proteins increased with an increase in the flavonoid concentration. When the concentration of the flavonoid fraction was 1/2 MIC, the extracellular nucleic acid concentration and soluble protein concentration of *B. cereus* were significantly different from those of the control group after 6 h of treatment. When the concentration of the flavonoid fraction was 1 MIC and 2 MIC, the concentration of the extracellular nucleic acid and soluble protein concentration was significantly different from that of the control group at 1 h and 4 h, respectively. Therefore, as the flavonoid concentration increased, the degree of damage to the bacterial permeability barrier increased, and the effluxed intracellular content of nucleic acids and proteins was elevated.

### 3.4 Antibiofilm activities of flavonoid fraction from *A. membranaceus* stems and leaves against pathogenic *B. cereus*

**FIGURE 6:**
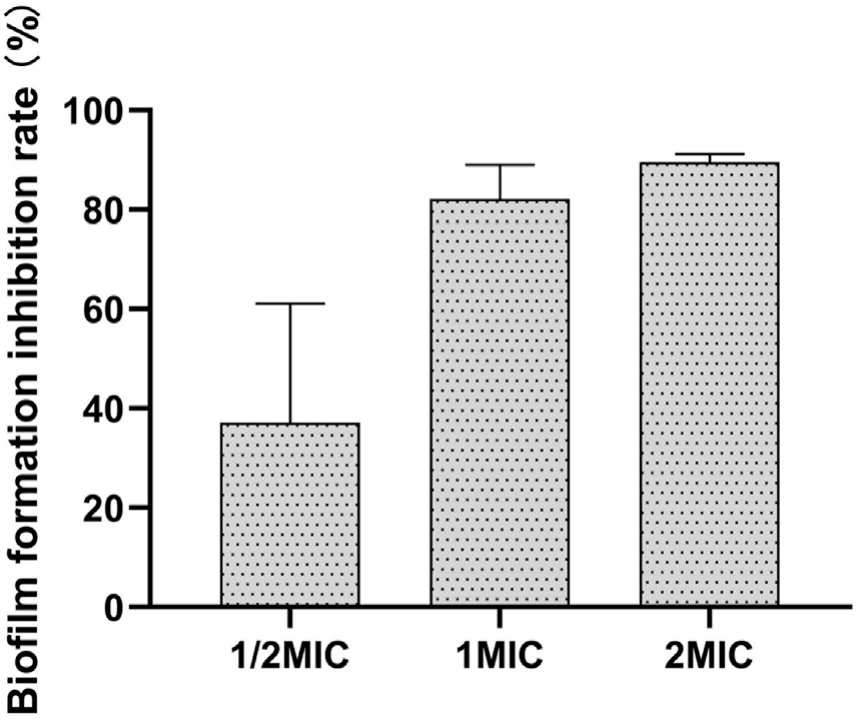
Antibiofilm activities of flavonoid fraction from *A. membranaceus* stems and leaves against *B. cereus*.

The results of different concentrations of the flavonoid fraction on *B. cereus* biofilm formation are shown in Figure 6. The flavonoid fraction at 1/2 MIC inhibited 37.11% biofilm formation of *B. cereus*. When the concentration of the flavonoid fraction was greater than 1 MIC, the inhibition rate of biofilm formation exceeded 80%. These results demonstrate that the flavonoid fraction could effectively reduce *B. cereus* biofilm formation.

### 3.5 Analysis of bacterial proteins by SDS-PAGE

**FIGURE 7:**
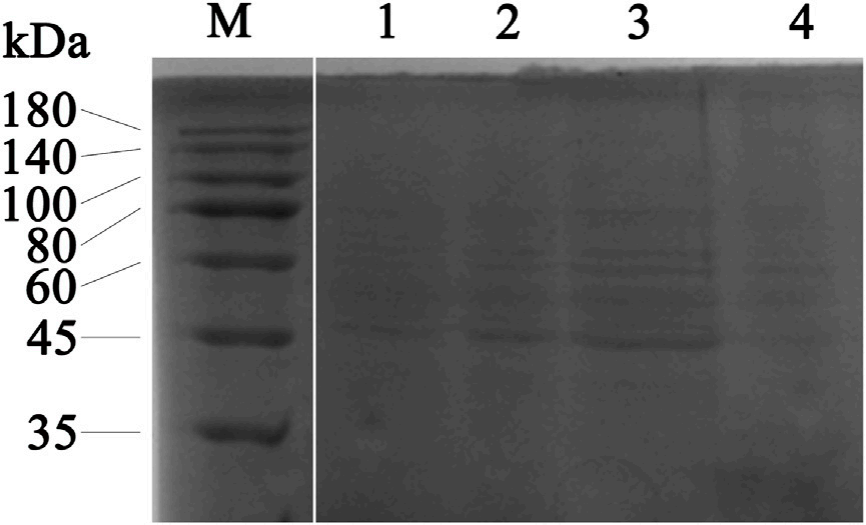
SDS-PAGE analysis of *B. cereus* proteins treated with concentrations of 1/2 MIC, 1 MIC, and 2 MIC of flavonoid fraction from *A. membranaceus* stems and leaves. Lane M = marker; lanes 1: without flavonoid treatment for 2 h; lanes 2, 3, and 4: treatment with 1/2 MIC, 1 MIC, and 2 MIC flavonoid fraction for 2 h, respectively. The gap is the figure splicing.

The effect of the flavonoid fraction from *A. membranaceus* stems and leaves on the soluble protein of *B. cereus* was explored by using SDS-PAGE, and the results are shown in Figure 7 the gap is the figure splicing. After treatment with the flavonoid fraction at 1/2 MIC, 1 MIC, and 2 MIC concentrations, the SDS-PAGE images showed significant changes in bacterial protein levels. The results showed that the *A. membranaceus* stem and leaf flavonoid fraction may have a certain promoting effect on the synthesis of bacterial proteins at low concentrations, but with an increase in the concentration of the flavonoid fraction, the bacterial protein levels of *B. cereus* became inhibited.

### 3.6 Virulence-associated genes

**FIGURE 8:**
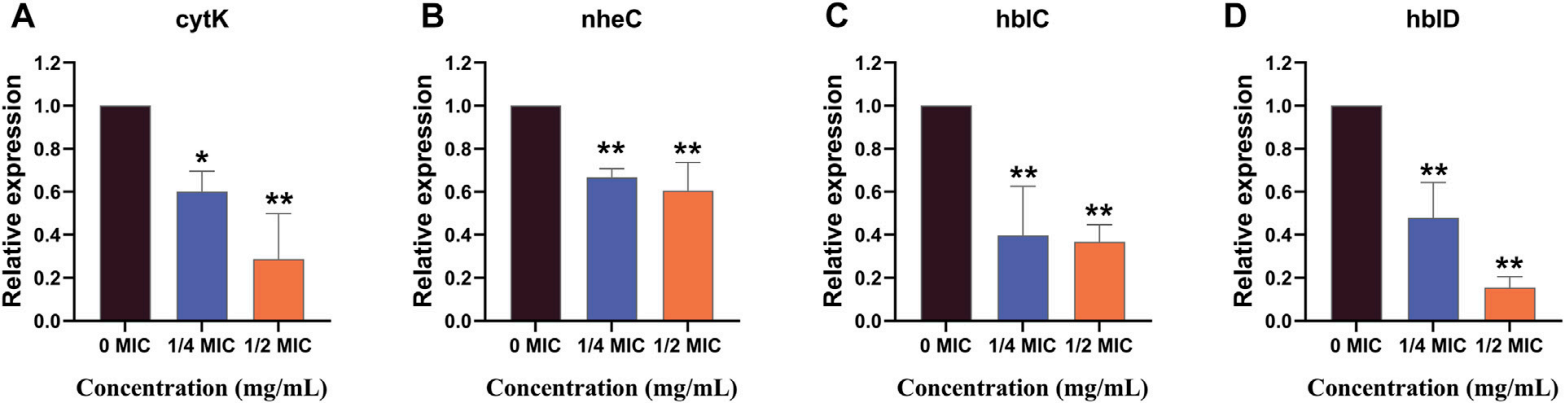
qRT-PCR analysis for expression of virulence-associated genes. “*” and “**” represent significance. (*cytK*: cytotoxin K gene; *nheC*: non-hemolytic enterotoxin gene; *hblC* and *hblD*: hemolysin BL gene).

The effects of the flavonoid fraction on the expression of *B. cereus* virulence genes are shown in Figure 8. The qRT-PCR results demonstrated that the relative expression levels of four virulence-related genes in *B. cereus* treated with 1/4 MIC and 1/2 MIC flavonoid fraction were significantly different from those in the control group. After 1/4 MIC and 1/2 MIC treatments, the relative expression of the cytotoxin K gene *cytK* decreased by 39.81% and 71.34%, respectively. When compared with the control group, significant differences were noted at the 0.05 and 0.01 levels, respectively. When the flavonoid concentration was 1/4 MIC, the relative expression level of the non-hemolytic gene *nheC* was 66.71% of the control group, and the relative expression levels of the hemolysin genes *hblC* and *hblD* were 39.66% and 47.89% of the control group, respectively. When the flavonoid concentration was 1/2 MIC, the relative expression levels of *nheC*, *hblC*, and *hblD* were 60.57%, 36.70%, and 15.41% of the control group, respectively, which are significantly different from those in the control group (*p* < 0.01).

### 3.7 Separation and purification of flavonoid fraction and HPLC-MS/MS analysis

**FIGURE 9:**
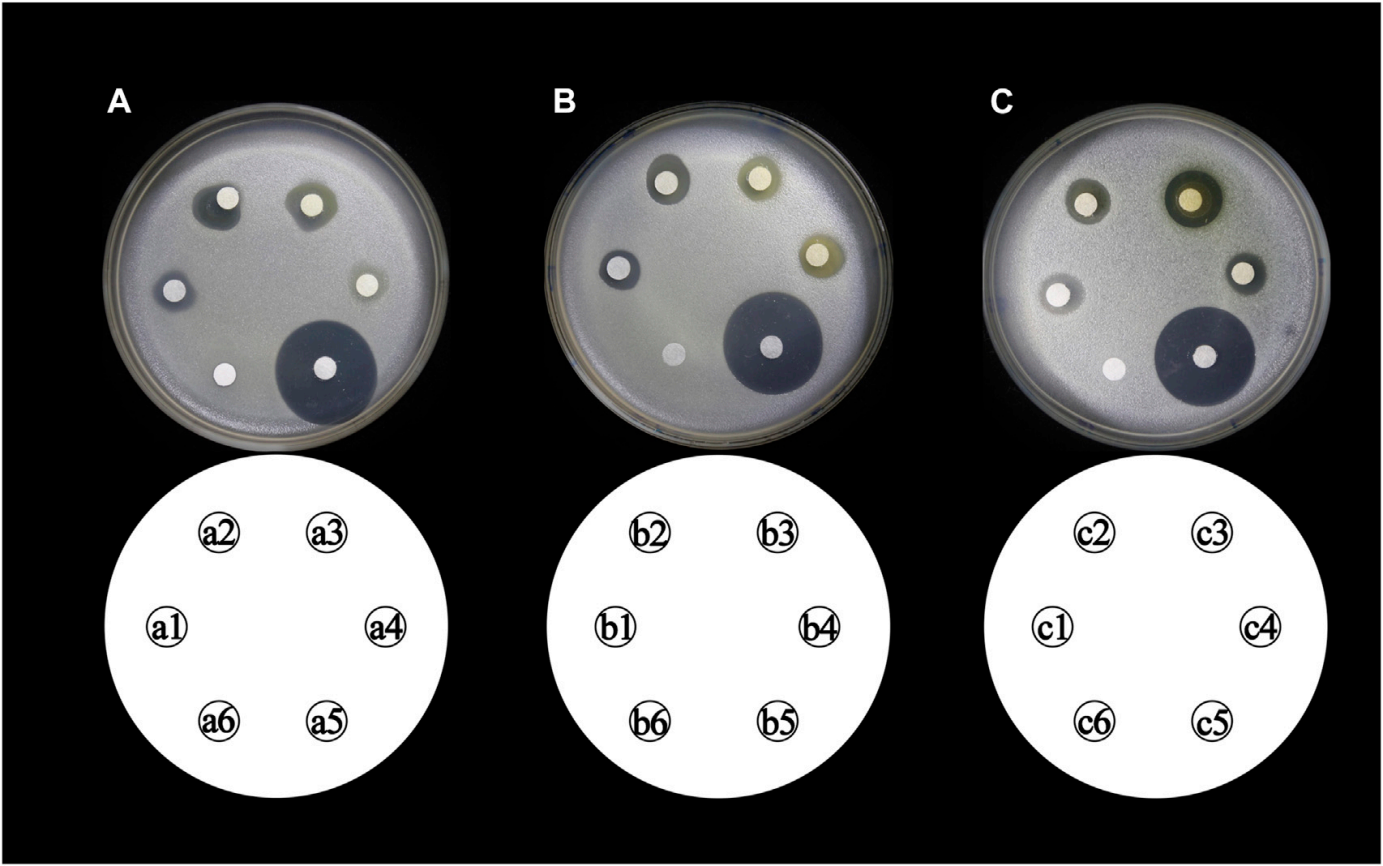
Inhibitory activity of different concentrations of flavonoid fraction from *A. membranaceus* stems and leaves against *B. cereus*. (a1, a2, a3, and a4 represent Frac. 30, 50, 70, and 95, respectively; b1, b2, b3, and b4 represent Frac. 50-1, 50-2, 50-3, and 50-4, respectively; c1, c2, c3, and c4 represent Frac. 50-2a, 50-2b, 50-2c, and 50-2d, respectively; a5, b5, and c5 represent positive controls chloramphenicol; and a6, b6, and c6 represent negative controls).

**FIGURE 10:**
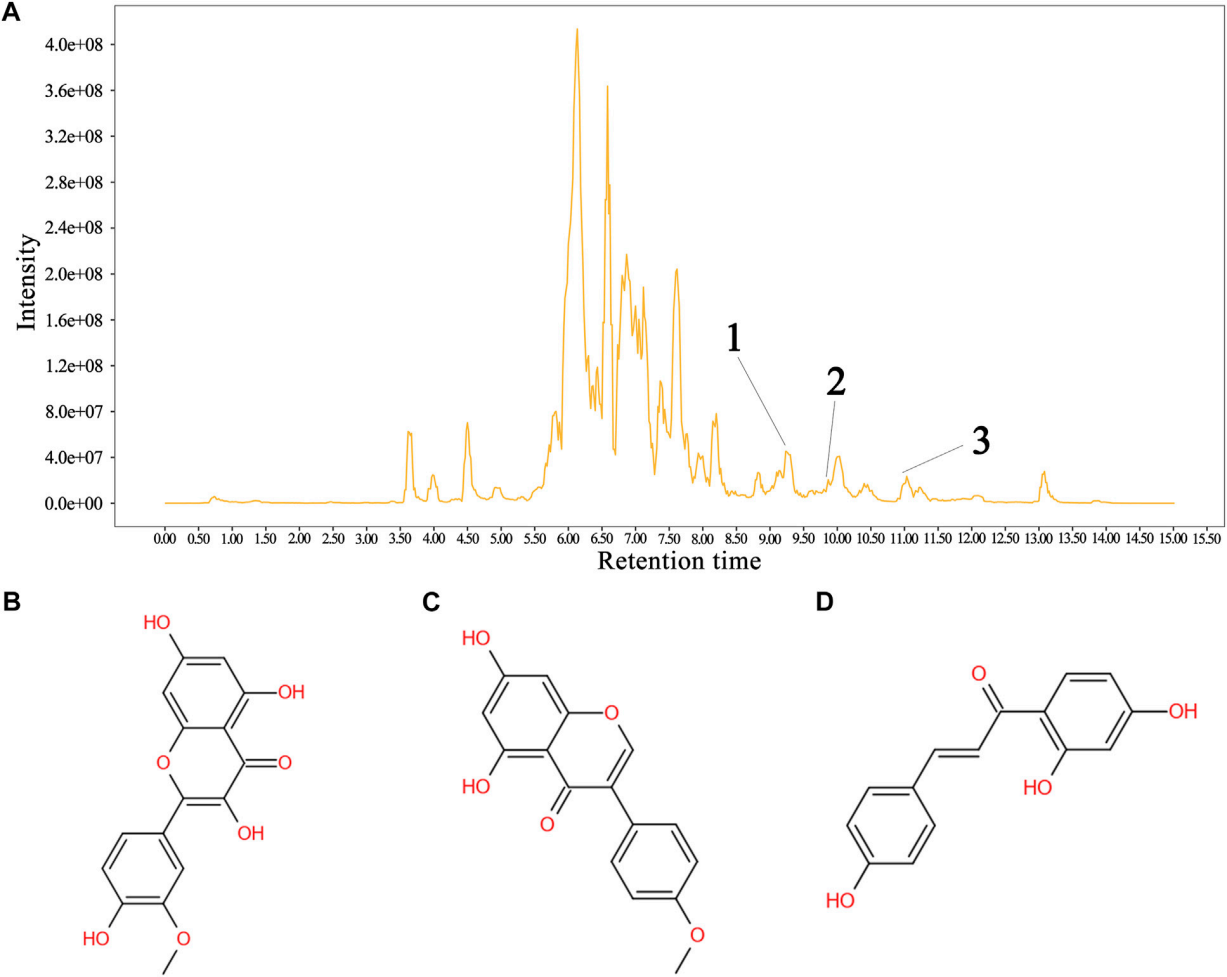
Sample HPLC/MS data for Frac. 50-2c and the structural formula of the metabolite. **(A)** chromatogram of Frac. 50-2c, **(B)** isorhamnetin, **(C)** biochanin A, and **(D)** isoliquiritigenin. In the HPLC profile: 1 = isorhamnetin, 2 = isoliquiritigenin, and 3 = biochanin A. Identification of the flavonoids is shown in the supplementary material (Table 2).

The total flavonoid fraction from *A. membranaceus* stems and leaves was isolated and purified by using AB-8 macroporous resin column chromatography, Sephadex LH-20 column chromatography, and TLC. First, the EAF was separated and purified by AB-8 macroporous resin, and four fractions (Frac. 30, 50, 70, and 95) were obtained by eluting with different concentrations of ethanol. Among them, Frac. 50 and 70 had the strongest inhibitory activity against *B. cereus* at 10 mg/mL (Figure 9A). Considering the low yield of Frac. 70, Frac. 50 was selected for further separation and purification. Second, Frac. 50 was separated and purified by using Sephadex LH-20 column chromatography to obtain four fractions (Frac. 50-1, 50-2, 50-3, and 50-4), of which Frac. 50-2 had the strongest inhibitory activity against *B. cereus* at 5 mg/mL (Figure 9B). Third, Frac. 50-2 was selected for further LH-20 column chromatography purification, and four fractions (Frac. 50-2a, 50-2b, 50-2c, and 50-2d) were obtained, of which Frac. 50-2c had the strongest inhibitory activity against *B. cereus* at 5 mg/mL (Figure 9C). The HPLC-MS/MS analysis of Frac. 50-2c showed that the main components were flavonoids (30.43%), phytohormone (23.32%), terpenoids (12.93%), phenolic acids (8.49%), and amino acids and nucleotides and their derivatives (8.48%). These components accounted for 83.65% of the total fraction, and other trace components accounted for the balance of the total fractions. Among the flavonoids, isorhamnetin, biochanin A, and isoliquiritigenin accounted for 45.32%, 18.01%, and 7.33%, respectively. The chromatogram is shown in Figure 10.

### 3.8 Verification test and molecular docking of isoliquiritigenin

**FIGURE 11:**
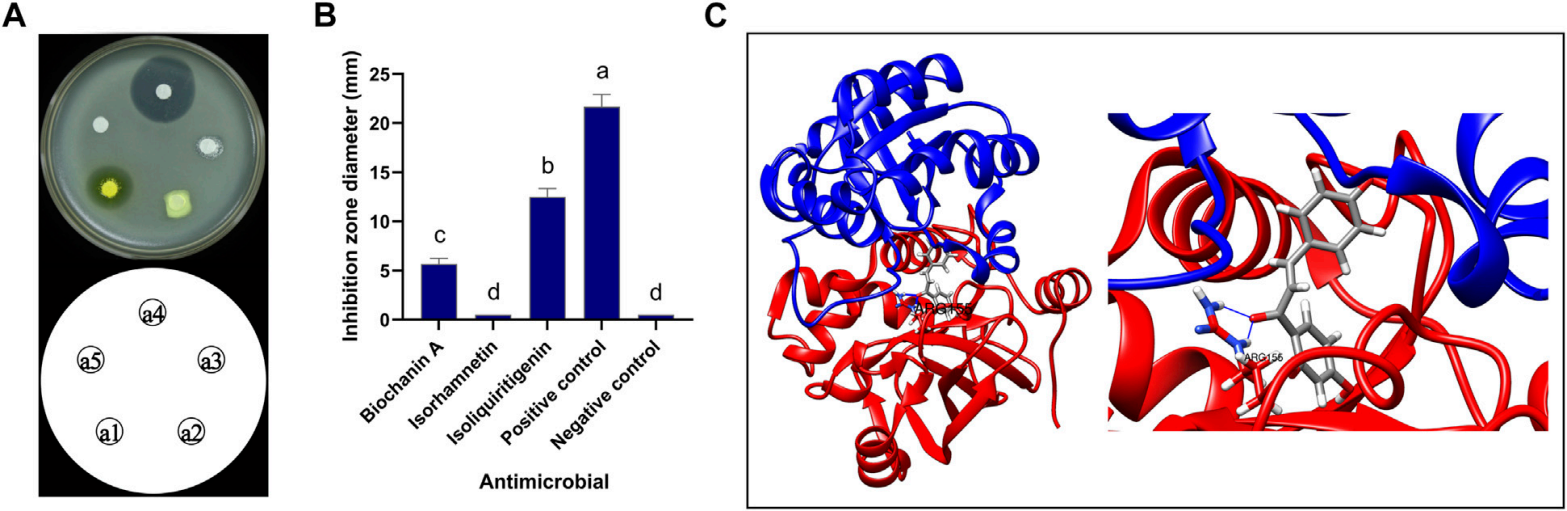
Antibacterial activity of isoliquiritigenin and 3D docking model with FtsZ. **(A)** Antibacterial activity of three components against *B. cereus*: a1: isoliquiritigenin, a2: isorhamnetin, a3: biochanin A, a4: positive control, and a5: negative control. **(B)** Inhibition zone diameter: a: the inhibition zone diameter of positive control was significantly different from that of isoliquiritigenin, biochanin A, isorhamnetin, and negative control; b: the inhibition zone diameter of liquiritigenin was significantly different from that of positive control, biochanin A, isorhamnetin, and negative control; c: the inhibition zone diameter of biochanin A was significantly different from that of positive control, isoliquiritigenin, isorhamnetin, and negative control; d: the inhibition zone diameter of isorhamnetin and negative control was significantly different from that of positive control, isoliquiritigenin, and biochanin A. **(C)** 3D docking model of isoliquiritigenin and FtsZ.

The inhibitory activity of pure commercial isoliquiritigenin, pure commercial biochanin A, and pure commercial isorhamnetin against *B. cereus* is shown in Figure 11. The antimicrobial activities of the three substances were significantly different (*p* < 0.05). Isoliquiritigenin had the strongest antibacterial activity, followed by biochanin A, and isorhamnetin had the weakest antibacterial activity. The binding mode of isoliquiritigenin with FtsZ is shown in Figure 11C, and the binding energy between the two was −8.2 kcal/mol. The binding site of isoquercitrin with FtsZ formed a suitable spatial complementarity that created two hydrogen bonds. The first oxygen atom formed two hydrogen bonds with two H atoms of Arg155 (B chain).

## 4 Discussion

With the prohibition and restriction of antibiotic additives in the world, plant-derived feed additives have attracted increasing attention. Feed products of traditional botanical drugs based on natural resources have become a hotspot in the study of antibiotic substitutes for feed products because of their immune enhancement, antimicrobial activity, digestion, and growth promotion (Yin, 2018; Ding et al., 2019; Guo et al., 2020). Using *A. membranaceus*, a traditional Chinese botanical drug, this study aimed to evaluate the antibacterial activity of its stem and leaf materials and conduct activity-guided separation and purification.

Antibiotic feed additives prevent subclinical infection by regulating animal intestinal flora. Therefore, antibacterial activity should be a prominent feature of plant-derived feed additives (Gaskin et al., 2002; Suresh et al., 2017; Jambwa et al., 2021). The antibacterial test of *A. membranaceus* stem and leaf extracts confirmed that *A. membranaceus* stem and leaf flavonoid fraction had different degrees of inhibition on the test strains, and the inhibitory activity on gram-positive bacteria was greater than that on gram-negative bacteria. In addition, this study found that the flavonoid fraction from *A. membranaceus* stems and leaves could destroy the cell wall and cell membrane of *B. cereus*. The cell wall and cell membrane are important components to maintain the normal shape of bacteria and control cell functions, so the flavonoid fraction from *A. membranaceus* stems and leaves have antibacterial and bactericidal effects that can destroy the permeability and integrity of cell walls and membranes (Yan et al., 2021). The results of the SDS-PAGE test showed that the protein in the supernatant of *B. cereus* cells was significantly reduced, which also indicates that the integrity of the cell membrane was damaged. These observations are similar to the results obtained for sugarcane bagasse extract against *S. aureus* and polyphenols from sugar beet molasses against *S. aureus*, *L. monocytogenes*, *E. coli*, and *S. typhimurium* (Zhao et al., 2015; Chen et al., 2017).

Biofilms are organized bacterial populations attached to the surfaces of living or inanimate objects and wrapped by bacterial extracellular macromolecules. When compared with planktonic bacteria, bacteria in biofilms are more resistant and virulent due to their high resistance to bad environments, antibiotics, and host immune defense mechanisms. The existence of biofilms not only provides the intracellular environment required for cell life activities but also serves as a medium for material transport, information transfer, and energy conversion between cells and cells and between cells and substrates. Therefore, infection with biofilm-forming pathogenic microorganisms may cause severe symptoms and even death (Fisher et al., 2010; Kostakioti et al., 2013; Clinton and Carter, 2015; Kumari and Sarkar, 2016). This study showed that the flavonoid fraction from *A. membranaceus* stems and leaves could effectively inhibit and reduce the formation of *B. cereus* biofilm. The toxicity of *B. cereus* is related to its virulence factors, such as ribosomal polypeptide synthase gene (*NRPS*), hemolysin BL gene (*hb1A*, *hb1B*, *hb1C*, and *hb1D*), non-hemolytic enterotoxin gene (*nheA*, *nheB*, and *nheC*), enterotoxin FM gene (*entFM*), enterotoxin T gene (*bceT*), and cytotoxin K gene (*cytK*) (Chen et al., 2020). Hbl, nhe complex, and cytK protein are considered the main virulence factors of *B. cereus* toxicity (Tewari et al., 2015). Hbl and nhe complexes combine with the cell membrane to cause cell osmotic lysis, and cytK is cytotoxic due to its pore-forming ability in cell membranes (Beecher and Wong, 1994; Toril et al., 2010; Kumari and Sarkar, 2016). This study demonstrated that the flavonoid fraction from *A. membranaceus* stems and leaves could downregulate the expression of the *cytK*, *nheC*, *hblC*, and *hblD* genes, thereby reducing the possibility of disease deterioration.

Bacterial cell division is a complex biological process regulated by multiple proteins, which includes accurate identification of division sites, localization of Z-ring, and coordinated contraction of the inner membrane and cell wall. The filamentous temperature-sensitive mutant Z (FtsZ) serves as a scaffold for recruiting downstream proteins, which persists throughout the division process. As the skeleton of the formation, location, and shape of the division membrane, it forms the cell wall after the mature formation of the division body and finally shrinks and divides into two daughter cells (Lock and Harry, 2008; Meier and Goley, 2014; Haeusser and Margolin, 2016; Huang et al., 2021). FtsZ is the most characteristic and conserved bacterial division protein, which is the key part of cell division in most bacteria. Studies have shown that the existence and activity of FtsZ play an important role in bacterial cell division and survival (Dai and Lutkenhaus, 1991; Tripathy and Sahu, 2019). In this study, molecular docking showed that isoliquiritigenin could combine with FtsZ to form a stable structure through hydrogen bond interaction to inhibit the activity of *B. cereus*.

Traditional botanical drug feed additives guided by the traditional theory of traditional botanical drugs have become an important direction for the development of feed products in recent years due to their effects of improving immunity; promoting growth; improving the quality of livestock and poultry products; and preventing bacterial infections, inflammation, and stress (Guo et al., 2020). In China, farmers’ recognition rate of traditional botanical drugs as an alternative to antibiotics is as high as 90%, and the popularization rate of traditional botanical drug feed products in meat and poultry farms is 95% (Guo et al., 2020). With the large-scale use of traditional botanical drug feed products in the feed industry, there will be a trend of tightening resources and competing with people for drugs in the future. In addition, with the continuous growth of traditional botanical drug feed products in the livestock and poultry breeding industry, resource demand will increase yearly, and raw material costs and agricultural and livestock products will show an upward trend. Therefore, the raw materials of feed products of traditional botanical drugs with large resource reserves and low production costs should be screened to achieve sustainable industrial development in the future. In the production and processing of *A. membranaceus*, the annual aboveground biomass is huge. However, the current *A. membranaceus* industry still belongs to the traditional production mode of mass production, mass consumption, and mass abandonment. The efficiency of resource utilization and industrial economic benefits are relatively low. A large amount of by-products and waste generated in the industrialization process is discharged as waste or simply converted into low value–added products, and the resource value has not been effectively released. In this study, the aboveground waste produced during the industrialization of *A. membranaceus* was used as raw material to analyze its antibacterial activity. The results showed that the flavonoid fraction from *A. membranaceus* stems and leaves had obvious inhibitory activity against *B. cereus*, which causes cow mastitis. Thus, *A. membranaceus* stems and leaves can be used as a potential alternative raw material for feed antibiotics to promote the healthy development of animal husbandry and realize further development and utilization of *A. membranaceus* resources.

## Data Availability

The original contributions presented in the study are included in the article/Supplementary Material; further inquiries can be directed to the corresponding authors.
