# Supplementary material for: Inhibitory activity of flavonoids fraction from Astragalus membranaceus Fisch. ex Bunge stems and leaves on Bacillus cereus and its separation and purification
Source: Front Pharmacol. 2023 Jul 3;14:1183393. doi: 10.3389/fphar.2023.1183393 (PMC10395332; doi:10.3389/fphar.2023.1183393)
Supplement: Supplementary file 1 [file DataSheet1.PDF]

Supplemental Table 1. the strain and molecule used in this study.

Strain/Molecule	Number	Strain/Molecule	Number
Bacillus cereus	CICC 21261	*Staphylococcus aureus*	CICC 21600
Escherichia coli	CICC 10389	*Shigella dysenteriae*	CICC 23829
Shigella flexneri	CICC 21534	*Cronobacter sakazakii*	CICC 21544
Salmonella enterica subsp. Enterica	CICC 21513		
FtsZ	PDB (4XSG)	isoliquiritigenin	PubChem (961-29-5)

Supplemental Table 2. HPLC-MS/MS analysis of flavonoids fraction from *A. membranaceus* stems and leaves.

No.	Compound	MW	q1	q3	Relative content (%)	RT (min)
1	Isorhamnetin	316.056	317.1	302.1	13.788%	9.23
2	Biochanin A	284.067	285.3	269.3	5.482%	11.21
3	Isoliquiritigenin	256.07	256.9	211	2.234%	9.73
4	Maltol	126.03	127	43	1.795%	0.9
5	Procyanidin B1	578.14	579.5	426.8	1.065%	4.03
6	3',4',7-Trihydroxyisoflavone	270.05	271.1	109	1.039%	8.98
7	Sciadopitysin	580.14	581.1	563.1	0.687%	12.82
8	Icariin	676.24	677.1	531.1	0.665%	8.12
9	2',6'-Dihydroxy 4'-methoxydihydrochalcone	272.10	273.1	105.1	0.519%	10.72
10	Pinobanksin	272.07	273.1	91.1	0.519%	8.14
11	Astragalin	448.10	449	286.9	0.481%	6.52
12	Liquiritin	418.13	417	254.9	0.289%	6
13	Protocatechualdehyde	138.03	138.9	120.8	0.229%	4.2
14	Isorhamnetin-3-O-nehesperidine	624.17	625.5	478.8	0.193%	6.02
15	6,8-Diprenylnaringenin	408.19	409.2	69.1	0.171%	12.62
16	Silibinin	482.12	483.5	464.8	0.148%	8.65
17	Protocatechuic acid;protocatechuic acid	154.03	153.05	108.95	0.125%	3.43
18	Genistin	432.11	433.3	270.8	0.125%	6.62
19	Tectochrysin	268.07	269	253.9	0.087%	11.8
20	Homoorientin	448.10	449	430.9	0.079%	5.43
21	Genistein	270.05	271.2	242.8	0.061%	8.93
22	Pyrocatechol	110.04	109	90.9	0.059%	4.09

23	Diosmetin	300.06	301.3	285.8	0.059%	9.15
24	Cajanol	316.09	317.1	123	0.059%	12.05
25	Schaftoside	564.15	565.5	546.9	0.048%	6.35
26	Plantagoside	466.11	467.1	287.1	0.046%	6.85
27	Chrysin	254.05	255.2	152.8	0.036%	10.94
28	Cynaroside	448.10	449	286.9	0.034%	6.1
29	Tectorigenin	300.06	300.9	285.5	0.031%	9.03
30	Apiin	564.15	565.5	432.9	0.023%	6.46
31	Tiliroside	594.14	595	308.8	0.020%	7.92
32	Homoferreirin	316.09	317.1	153	0.017%	8.09
33	Pinocembrin	256.07	257.3	152.8	0.016%	11.09
34	Tricin	330.07	331.1	313.1	0.015%	9.31
35	Nobiletin	402.13	403.4	387.8	0.014%	11.03
36	Apigenin	270.05	270.9	163	0.013%	8.9
37	Osajin	404.16	405.2	349.1	0.013%	11.06
38	(-)-Epigallocatechin	306.07	306.9	180.9	0.013%	4.12
39	Physcion 1-O-beta-D-glucoside;Sissotrin;Physcion 8-O-beta-D-monoglucoside	446.12	447.1	267.1	0.012%	6.7
40	(-)-alpha-Narcotine	413.15	414.2	220.1	0.010%	12.1
41	Eriodictyol	288.06	288.9	163	0.010%	7.9
42	Daidzin	416.11	416.9	255	0.010%	5.45
43	Aromadendrin	288.06	289.1	107	0.009%	11.21
44	Quercetin	302.04	300.9	272.9	0.008%	8.07
45	Isoquercitrin	464.10	465.4	302.8	0.008%	6.06
46	6,7,4'-Trihydroxyisoflavone	270.05	271.1	105	0.008%	10.76

47	Ononin	430.13	431.4	430.9	0.008%	7.3
48	Sakuranetin	286.08	287.1	105	0.008%	9.94
49	Sinensetin	372.12	373	357.4	0.007%	10.34
50	Pedalitin	316.06	317.1	109	0.006%	12.08
51	Genkwanin	284.07	284.9	269.9	0.006%	11.1
52	4',7-Isoflavandiol	242.09	243.1	123	0.006%	11.35
53	Baicalein	270.05	269	238.9	0.006%	9.38
54	Hydroxysafflor yellow A	612.17	613.2	595.2	0.005%	12.59
55	7-Hydroxyflavanone	240.08	241.1	101	0.005%	4.81

Note: “MW” represents molecular weight; “q1” represents ratio parent ion mass charge; “q3” represents ratio daughter ion mass charge; “RT” represents retention time (min).
